# Mechanical property measurements enabled by short-term Fourier-transform of atomic force microscopy thermal deflection analysis

**DOI:** 10.3762/bjnano.16.136

**Published:** 2025-11-06

**Authors:** Thomas Mathias, Roland Bennewitz, Philip Egberts

**Affiliations:** 1 Department of Mechanical and Manufacturing Engineering, University of Calgary, 2500 University Drive NW, Calgary, AB, T2N 1Y6, Canada; 2 INM-Leibniz Institute for New Materials, Campus D2 2, 66123 Saarbrücken, Germany

**Keywords:** atomic force microscopy, contact resonance, highly oriented pyrolytic graphite (HOPG), mechanical property measurements, surface science

## Abstract

Contact resonance atomic force microscopy (CR-AFM) has been used in many studies to characterize variations in the elastic and viscoelastic constants of materials along a heterogeneous surface. In almost all experimental work, the quantitative modulus of the surface is calculated in reference to a known reference material, rather than calculated directly from the dynamics models of the cantilever. We measured the cantilever displacement with very high sampling frequencies over the course of the experiment and captured its oscillations that result from thermal energy. Using short-term Fourier transformations, it was possible to fit the thermal resonance peak of the normal displacement to track the frequency and Q-factor of the cantilever during an experiment, using a similar process to that used to calibrate the normal bending stiffness of cantilevers. With this quantitative data, we have used the dynamic mechanics models relating the contact stiffness of the tip/cantilever pressing into a surface with the oscillation frequency of the cantilever and show that they did not accurately model the experiment. Several material combinations of tip and sample were examined; tip size and cantilever stiffness demonstrate that existing models cannot capture the physics of this problem. While concrete solutions to use analytical models to interpret CR-AFM data have not been found, a possible solution may include revisiting the analytical model to capture a potentially more complex system than the current model, improved matching the cantilever/sample stiffness to obtain a larger variation in contact stiffness with frequency, or investigating the use of higher-order modes that may achieve this improved match.

## Introduction

Atomic force microscopy (AFM) has become an indispensable tool for imaging the surface topography on a variety of surfaces [1]. Since the invention of the AFM [2], several other modes of AFM have been developed, including friction force microscopy [3], tapping mode AFM [4], and contact resonance AFM (CR-AFM) [5], each providing unique advantages or insights into a surface and the materials that comprise it. Alongside the developments of the experimental technique, there have been a number of modeling techniques created that can be used to bring physical values or interpretation to the data that is collected by the AFM, allowing operators of the technique to compare their measurements across fields [6].

CR-AFM is a technique that was established in 2008, allowing for the measurement of mechanical properties (elastic modulus and viscoelastic modulus) of surfaces [5]. It is particularly useful for the measurement of heterogeneous surfaces, characteristic of composite and biological materials, where understanding the interplay between microstructure and mechanical properties of the constituent materials is critical for the performance of the overall structure. Analytical models for interpreting the vibrational modes of cantilevers were developed prior to the invention of the technique [7–8]. This model or variations of it are often presented in manuscripts to explain the interpretation of experimental data, but are not used to bring physical meaning to the experimental data. Instead, in almost every example in the literature, the frequency variation is normalized to what is measured on a surface having known mechanical properties [5,9–10].

Alongside the development of CR-AFM and the analytical models used to describe the technique, spectral analysis of the thermal motion in the deflection of AFM cantilevers has shown promise as a lower-cost, less equipment-intensive mechanism to access the dynamic and time-evolving oscillatory characteristics of the cantilever [11–16]. In these techniques, the cantilever deflection signal is acquired at rates several times greater than the first normal resonant frequency (typically greater than 1 MHz) for several seconds, as the cantilever is approached, and the tip is pressed against, and finally removed from, a surface. In a significant number of studies, a Fourier transform, or transformation of the time-based AFM deflection into the frequency domain is conducted. In the majority of studies examining the thermal oscillations of AFM cantilevers, the cantilever’s displacement is measured for a specific length of time and then converted to frequency space over the entire length of the measurement [14–16]. This calculation results in a single measurement of the cantilever’s oscillation frequency and other oscillation parameters, but can also result in high frequency/spectral resolution that can allow for the determination of quantifiable results for parameters such as elastic modulus or viscoelastic properties when the duration of the cantilever’s thermal motion was measured for sufficiently long times [15]. However, the drawback of this type of measurement is that, with such infrequent measurements of the cantilever’s oscillation characteristics, it is difficult to measure mechanical properties of heterogeneous surfaces as the cantilever is scanned over the surface, or to measure how mechanical properties of the surface evolve with time as the cantilever is pressed against the surface. Furthermore, the analysis assumes that the cantilever’s oscillation characteristics are static over the measurement period, which often is not the case. To solve this issue, wavelet transformations of the AFM cantilever’s deflection signal have been conducted, allowing for several frequency spectra at defined time intervals to be calculated over the course of the experiment [11–13].The drawback to most wavelet transforms applied to analyze AFM thermal deflection signals is that these measurements suffer from insufficient spectral resolution, which limits the ability to accurately quantify cantilever oscillation characteristics, as well as making it difficult to obtain quantitative measurements from the frequency of the AFM cantilever’s bending mode.

In this manuscript, we bring together the analytical models that describe cantilever oscillations in AFM experiments where a tip is oscillated and pressed into contact with a solid surface [7–8] with the spectral analysis of the thermal motion of the cantilever using short-term Fourier transforms (STFTs). Similar to wavelet transforms, STFTs allow one to calculate the time-varying spectra of the cantilever’s deflection signal over the measurement time with a simpler way of controlling the spectral/frequency resolution, supporting the end goal of quantifiable mechanical property data. Here, we observe the thermal oscillations of the AFM cantilever rather than an externally excited cantilever. An advantage to observing and analyzing the thermal oscillations of the cantilever is that the oscillations of the cantilever have sub-angstrom amplitudes regardless of if the tip is in contact with the sample or far from the surface. When the tip is in contact with the surface, these small oscillations of the AFM cantilever are much smaller than atomic bonds in our materials, which then can be interpreted as a small perturbation to the system that is examined. We also avoid disturbance of the medium surrounding the sample, as occurs with piezoacoustic excitation of the cantilever, without requiring expensive modification of our existing AFM system. Finally, by avoiding the use of a phase-locked loop to track the frequency of the cantilever oscillation and rather using STFTs to calculate time-varying frequency spectra, we are able to monitor the oscillation of the cantilever as it transitions from free out-of-contact to in-contact, changing the oscillation mode of the cantilever. Additionally, spectral analysis allows for the measurement and tracking of all resonant modes simultaneously, which would otherwise require a separate phase-locked loop for each mode to be tracked.

To examine and validate the use of spectral analysis of the thermal motion of AFM cantilevers as an alternative approach to CR-AFM, we conducted AFM experiments on well-characterized surfaces, such as highly ordered pyrolytic graphite (HOPG), using silicon cantilevers with integrated probes. To examine the time evolution of the AFM cantilever’s oscillatory modes during an experiment, STFTs, rather than wavelet transforms, of the thermal motion of the AFM cantilever were calculated. Once calculated, the resonant peak corresponding to the cantilever’s first oscillatory mode was fit, yielding the time-evolving parameters of the AFM cantilever be to tracked over the course of the experiment, such as the resonant frequency. CR-AFM models were used to determine the size of the tip–sample contact, assuming the relevant material parameters of the system examined. Finally, the same experiment and data analysis was performed with other substrates and AFM tip materials to further explore the analytical CR-AFM models.

## Methods

### Experimental design

An Agilent Keysight 5500 AFM was used in all experiments with measurements conducted under ambient laboratory conditions of 20–40% humidity. Four samples were analyzed in the experiments, namely, a silicon wafer, freshly-cleaved HOPG, poly(ethylene oxide) (PEO), and polydimethylsiloxane (PDMS). The mechanical properties of these samples are provided in Table 1.

**Table 1:** Mechanical properties of the examined samples. Values for silicon, HOPG, and platinum are from [17], [18], and [19], respectively. The values for PEO and PDMS were measured using a Hysitron Premier Nanoindenter.

Material	Young’s modulus (GPa)	Poisson’s ratio

silicon	160	0.3
HOPG	20	0.25
platinum	140	0.38
PEO	*0.22 ± 0.03*	0.5
PDMS	*0.0025 ± 0.0002*	0.45

Silicon wafers were ultrasonicated in acetone and ethanol for 10 min each. HOPG samples were cleaved using the Scotch tape method within 30 min of beginning an experiment. Finally, the PEO and PDMS samples were not surface-treated following their polymerization/deposition. The topography of the surface was measured before acquiring a force-versus-distance measurement to ensure that these measurements were acquired on clean and flat regions of the substrate. To observe how the resonant frequency of the AFM cantilever changes as the attached tip is pressed against a substrate, force-versus-distance measurements were conducted. In these measurements, the sample was moved up and down at a rate of approximately 100 nm·s^−1^ while recording the cantilever deflection over the course of the measurement. In addition to the AFM’s own control software measuring the deflection of the cantilever and moving the sample during the experiment, the cantilever deflection was measured by a National Instruments BNC box (NI-USB-6341) via an unfiltered connection direct from the photodetector at a sampling rate of 2.0 MHz and for a duration of 1 s of the experiment, unless otherwise noted. The data from this instrument will be referred to in the paper as the “high-sample rate” data.

Three types of uncoated cantilevers were used all experiments, that is, soft cantilevers with an integrated tip (Nanosensors PPP-CONT), soft tipless cantilevers (Nanosensors TL-CONT), and harder cantilevers with an integrated tip (Nanosensors PPP-NCL). The soft cantilevers have a nominal stiffness in the normal bending direction of 0.2 N·m^−1^, and the hard cantilevers have a nominal stiffness of 40 N·m^−1^. For each cantilever used, the spring constant of the cantilever in the normal bending direction was determined through the Sader method [20], with the plan-view dimensions and the setback of the tip from the end of the cantilever measured in an optical microscope. To convert the voltage signal measured by the photodetector, the slope of the force versus distance curve generated from the manufacturer’s software was determined, having a unit of volts per meter. Four different tip materials were used in experiments, namely, conventional silicon cantilevers (Nanosensors PPP-CONT), conductive diamond-coated probes (Nanosensors CDT-CONTR), platinum silicide-coated probes (Nanosensors PtSi-CONT), and borosilicate glass colloids (Sigma-Aldrich 440345-100G) attached to the tipless cantilevers (Nanosensors TL-CONT). The borosilicate glass colloids had a diameter of 8–11 μm and an elastic modulus of 60 GPa.

### Data analysis

Following completion of experiments, post processing of the high-sample rate data was performed. This data was windowed into segments of data having lengths of 2*^N^* in number of data points, with *N* ranging from 10 to 20. These windowed segments were convolved with the Hanning window to reduce spectral leakage. For each window, a Fourier transform was calculated and stored. Subsequently, for each window generated, the resonant peak of the first normal mode was fitted using Equation 1,


[1] $$A\left(f\right)=\frac{k_{\text{B}}Tf_{n}^{3}}{\pi Q_{n}D_{n}{\left(f^{2}-f_{n}^{2}\right)}^{2}+{\left(\frac{ff_{n}}{Q_{n}}\right)}^{2}}\cdot 10^{18}+y_{0},$$

where *f* is the frequency, *T* is the temperature, *k*_B_ = 1.3806 × 10^−23^ m^2^·kg·s^−2^·K^−1^ is Boltzmann’s constant, *Q_n_* is the quality factor of the cantilever for the *n*-th mode, *D_n_* is the stiffness of the *n*-th oscillation mode, and *y*_0_ is an offset value [21]. Fits of these resonant peaks using the non-linear least squares method yielded the parameters *f_n_*, *Q_n_*, and *D_n_*. To ensure accurate fits to the resonance peak, the window size *N*, impacting the frequency resolution (*f*_Δ_) of the calculated Fourier transform, was carefully chosen to ensure that β in Equation 2 was much larger than 1 [22]:


[2] $$\beta =\frac{\pi }{2Q_{n}}\frac{f_{n}}{f_{\Delta }}.$$

Fitting of the first resonant peak of the cantilever in contact with the surface during the force-versus-distance measurement thus provides the evolution of the *f*_1_, *Q*_1_, and *D*_1_ values as functions of time during the experiments. These values can be related to the displacement of the sample, force, or other parameters that are time-averaged over the window size.

### Analytical models of cantilever dynamics

**Figure 1:**
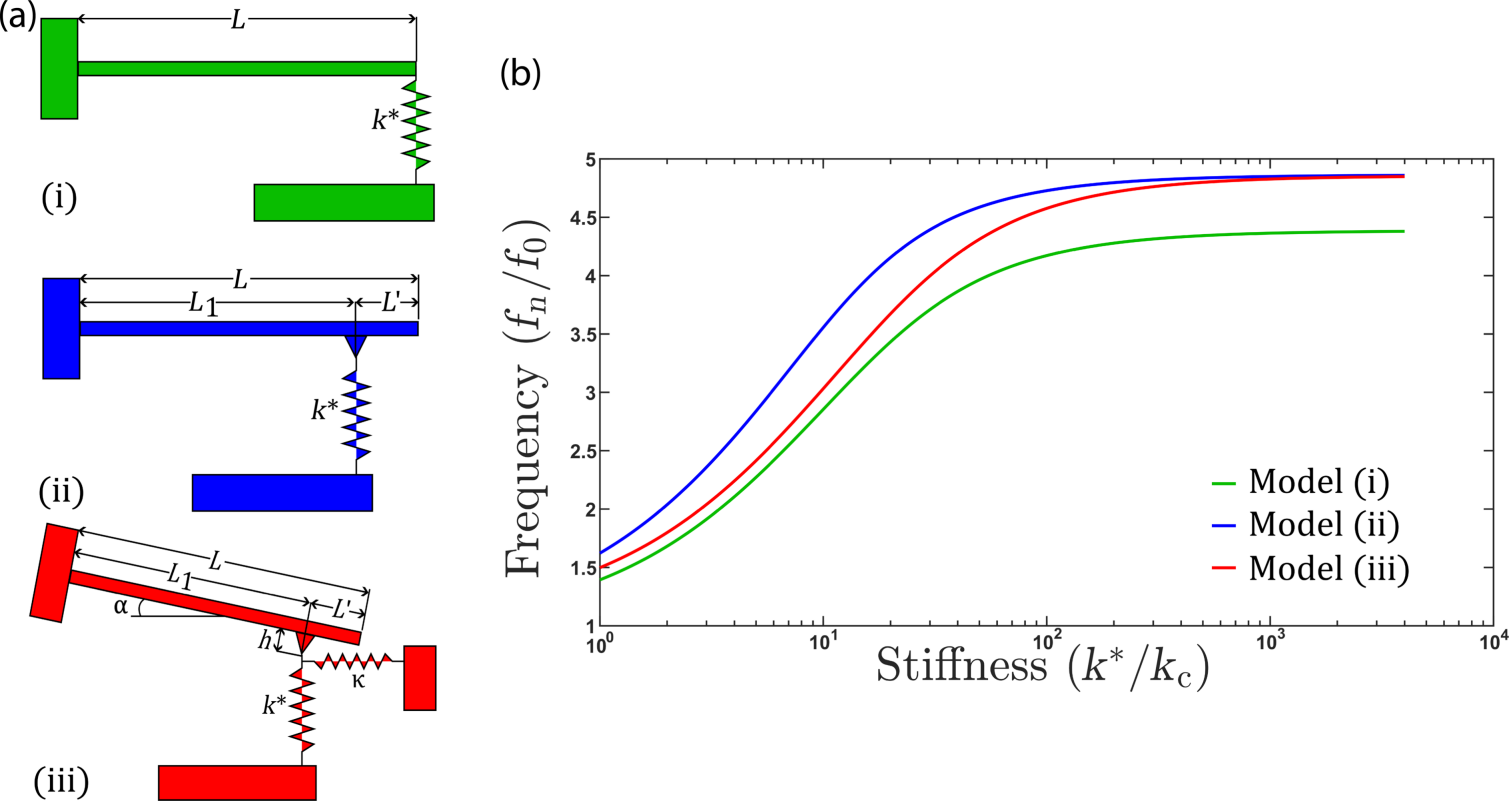
(a) Schematic diagrams of the cantilever models used in determining the dispersion curves to convert measured cantilever oscillation frequency to contact stiffness of the tip–sample contact. Three models are typically used. Model (i) shows the tip at the end of the cantilever, model (ii) shows the tip set back from the end of the cantilever, and model (iii) shows a cantilever tilted with respect to the surface and the tip set back from the end of the cantilever. *L* is the overall cantilever length, *L*^′^ is the distance that the tip is set back from the end of the cantilever, k* is the contact stiffness, α is the tilt angle of the cantilever with respect to the surface, *h* is the distance between the tip apex and the cantilever base, and κ = 8G*a ([23]) is the lateral stiffness of the tip–sample contact. (b) Dispersion curves providing a lookup table for the conversion of measured resonant frequency to tip–sample contact stiffness. Model (i) is shown in black, model (ii) in blue, and model (iii) in red.

Several analytical models of cantilever dynamics have been developed, with the basis of most models originating from the work by Rabe et al. [7], and are schematically shown in Figure 1a(i) and Figure 1a(ii). More advanced models that include the tilt angle of the cantilever relative to the surface [23], to better reflect the typical 12.5° or 22.5° angles of the cantilever relative to the surface, have been subsequently developed and are shown in Figure 1a(iii). To relate the oscillation frequency of the cantilever to the contact stiffness, equations of motion for the schematic have been developed in [7,23] and are provided in Supporting Information File 1 for reference. These equations are used to develop the dispersion curve shown in Figure 1b. The dispersion curve shows how the measured frequency changes as the contact becomes stiffer, which occurs in the previously described experiments when the tip is pressed against the surface with a larger normal force. Typically, CR-AFM experiments are conducted at a constant normal force (increasing the tip–sample contact size); thus, changes in the contact stiffness result from variations in the elastic modulus, *E*, along the surface. The relation between elastic modulus, contact size, and contact stiffness is found in Equation 3 [24],


[3] $$k^{*}=2aE^{*},$$

[4] $$\frac{1}{E^{*}}=\frac{1-\nu_{\text{sample}}^{2}}{E_{\text{sample}}}+\frac{1-\nu_{\text{tip}}^{2}}{E_{\text{tip}}^{*}},$$

where *a* is the size of the contact between the tip and sample, E* is the reduced elastic modulus defined in Equation 4, ν is the Poisson ratio of tip or sample, and *E* is the elastic modulus of tip or sample.

## Results and Discussion

**Figure 2:**
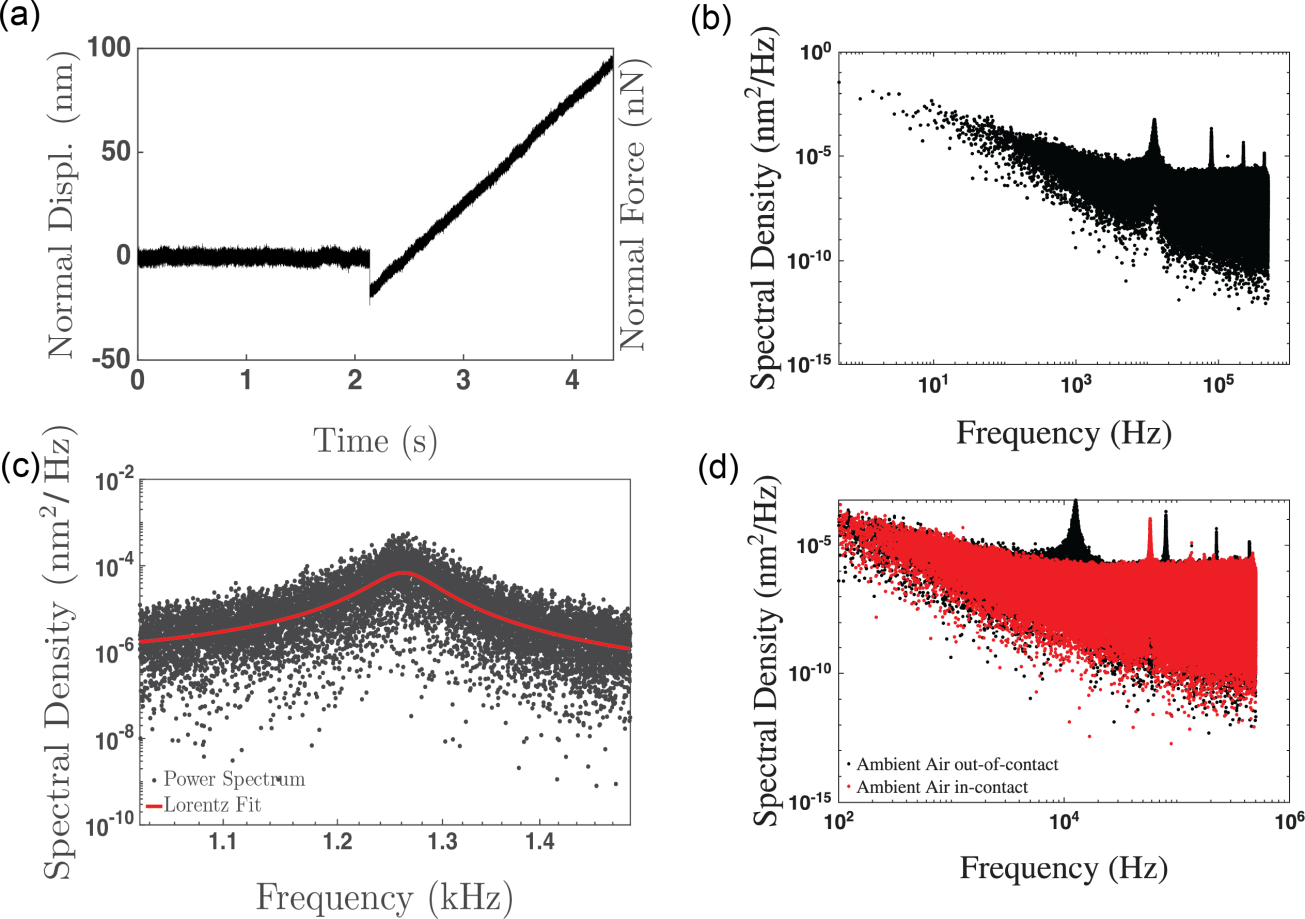
(a) Force-versus-displacement curve using the high-sample rate acquisition system. (b) Fourier transform of the out-of-contact portion of (a). (c) Fit (red line) of the first resonant mode peak (black squares) with Equation 1. (d) Fourier transform of the out-of-contact portion of (a) shown in black and the in-contact portion shown in red, highlighting the change in the resonant peak locations and shapes between these two stages of the measurement. Data was acquired at 1 MHz for approximately 4.5 s.

Figure 2a shows an exemplary force-versus-distance measurement acquired with the high-sample rate acquisition system for a soft silicon cantilever on a HOPG substrate. Both the normal force and the cantilever displacement values are shown as most AFM studies report normal force values, but the power spectrum calculation requires the cantilever displacement values. Figure 2b shows the calculated Fourier transform/power spectrum of the cantilever displacement in the out-of-contact portion of Figure 2a, that is, the data acquired from approximately 0 to 2 s of the experiment. The power spectrum clearly shows the first four oscillation modes of the cantilever, with the first oscillation mode having the largest amplitude. Figure 2c shows the quality of the fit obtained using Equation 1 to the first oscillation mode, yielding values of *f*_1_ = 12.627 ± 0.003 kHz, *Q*_1_ = 19.84 ± 0.20, and *D*_1_ = 25.67 ± 0.02 mN·m^−1^. We note that the fit value obtained from Equation 1 is not the same value as the one obtained using the Sader method (74.3 mN·m^−1^ for this cantilever in Figure 2) [20]. Similar observations were made for the other cantilevers used in the experiments conducted within this paper, with the difference between the value of *D*_1_ and the normal spring constant calculated using the Sader method ranging between a factor of 2 and 10. This difference is likely a result of the plan-view dimensions of the cantilevers having dimensions beyond the 10% variation of the manufacturer’s specifications, observed in other experiments we have conducted outside this study. Viscous damping from the ambient environment is not accounted for in Equation 1 and may also be responsible for a small percentage of the difference between the two calculations of the spring constants. However, our results highlight that the measurement of the cantilever’s plan-view dimensions and using these dimensions in the determination of the Sader spring constant or other calculations of the normal spring constant are important. Finally, it has been demonstrated that the Sader method can consistently show a difference compared with the thermal noise method used above, particularly for soft cantilevers as used in this study [25]. We take the Sader spring constant, which has been widely used in other studies and is less sensitive to variations in the calculated cantilever sensitivity [25], as the spring constant of all cantilevers in the calculations in subsequent sections of this manuscript.

Figure 2d shows two power spectra, the black spectrum calculated from the time ranging from 0 to 2 s, and the second in red from the time ranging between 2.5 and 4.5 s. These two spectra highlight the change in the location and shape of the normal resonant peaks for the cantilever from when the cantilever was out of contact to when it was in contact. We are able to estimate the values of the various modes, as Rabe et al. showed that the value of *f_n_*/(*k_n_L*)^2^ is a constant for the cantilever, which also allows us to distinguish between higher-order oscillatory modes of the cantilever and pinning of the free end of the cantilever [7]. With the first resonant peak out of contact having a center frequency of 12.62 kHz and using model (i) to estimate the location of subsequent resonant peaks, the expected second resonant mode of a free cantilever would be approximately 79.1 kHz, in contrast to an expected frequency of 55.3 kHz in the first resonant mode if the end of the cantilever was completely pinned. The measured value of the cantilever resonant frequency when the tip was pressed into the surface was 58.15 kHz, which is much closer to the expected value of pinned cantilever than the second resonant mode. Beyond identifying and fitting the first pinned mode of oscillation, it is also possible to observe several of the higher modes within the in-contact power spectrum compared with the out-of-contact spectrum. Finally, we note that the full width at half maximum increases slightly for the first oscillation mode when the cantilever makes contact with the surface, but shows significant scatter during the force curve measurement, making a statement regarding the variation of the Q-factor difficult with the present analysis technique.

**Figure 3:**
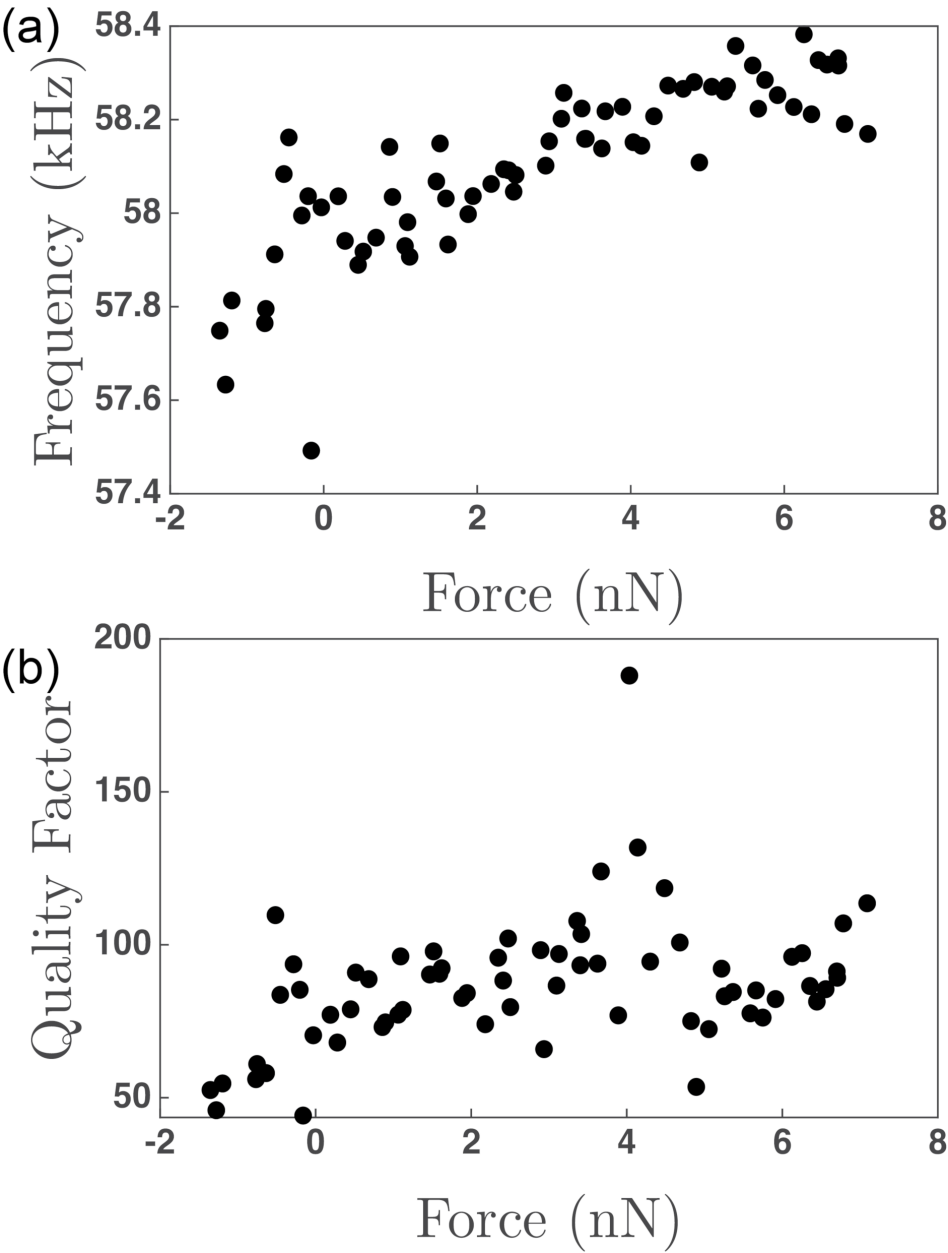
(a) Resonant frequency versus normal force determined from fits of the first normal resonant mode peak in the power spectra of the contact portion of Figure 2. (b) Q-factor (*Q*) versus normal force similarly determined from the power spectra of the contact portion of Figure 2. *N* = 17 in (a) and (b).

Figure 3a shows the variation of the frequency of the first normal mode as a function of normal force during the in-contact period of the force curve. A sub-linear variation is observed with increasing applied normal force. Figure 3b shows the variation of the quality factor with normal force, simultaneously determined with the frequency of the first normal oscillatory mode. Here, the variation in the Q-factor is less clear than for the resonant frequency. An initial increase is observed, which plateaus around 0 nN applied force. However, significant scatter in the Q-factor is observed, in particular compared with the variation in the frequency of the first normal oscillatory mode. Significantly more scatter is observed for the last fit parameter, *D*_1_, which, in the case of a free oscillation, represents the spring constant of the single-harmonic-oscillator mode. Additionally, the physical meaning of *D*_1_ is less clear when the tip/cantilever is in contact with the surface than when it is free. Thus, how *D*_1_ varies over the course of the experiment has been included in Supporting Information File 1, Figure S1 for completeness but is not further analyzed within the manuscript.

**Figure 4:**
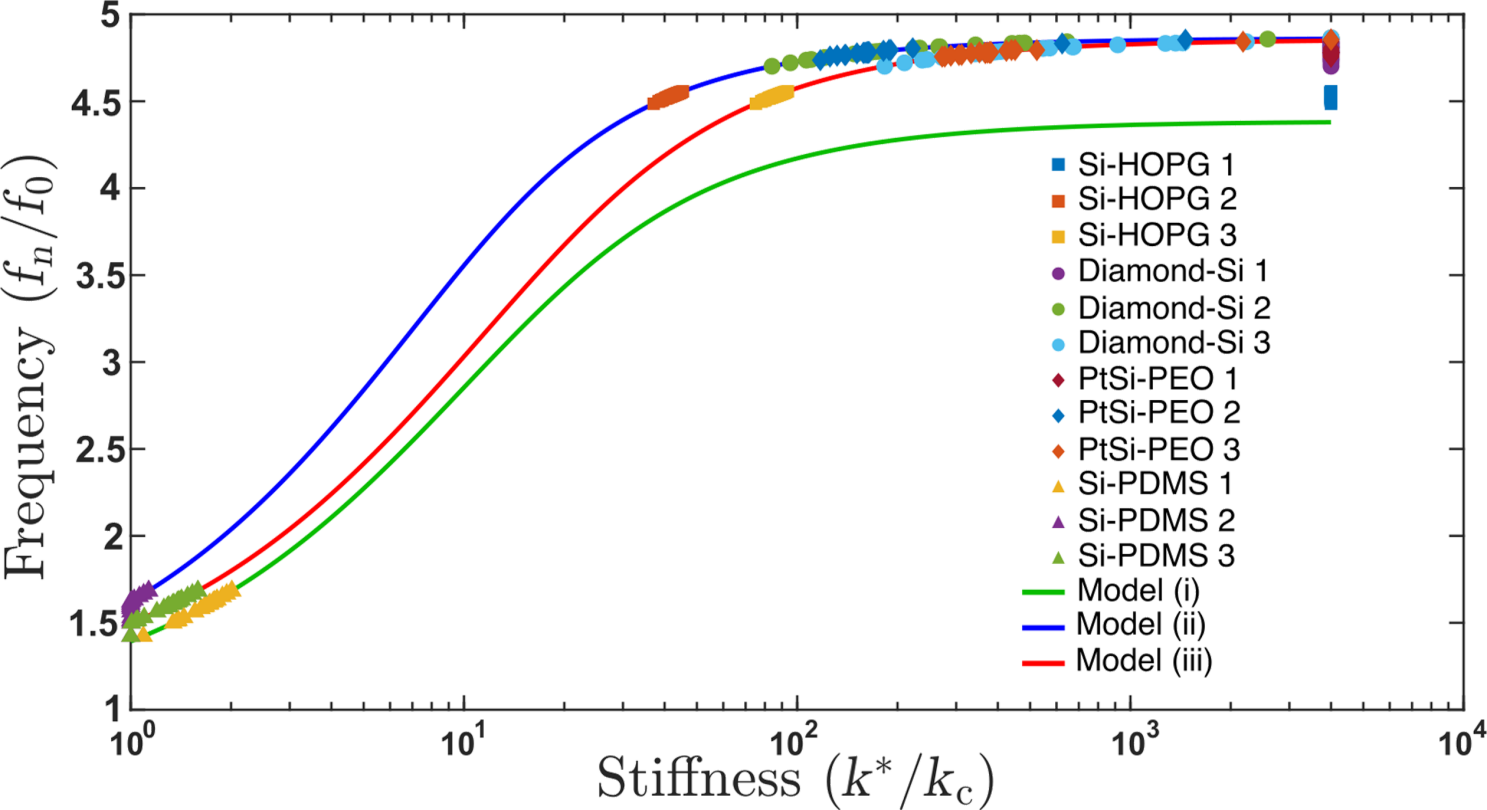
Experimental data for four sample combinations tested (silicon tips on HOPG, diamond-coated tips on silicon, silicon tip on PEO, and silicon tip on PDMS) plotted for the three cantilever models.

Figure 4 shows the dispersion curves generated for the three cantilever models, with the data obtained from all material combinations evaluated in this study in each of the models. For example, Figure 4 shows that for soft materials, such as the Si–PDMS combination (silicon cantilever and PDMS substrate), all three models can be used to translate the oscillation frequency variation into a contact stiffness. However, for harder materials, such as Si–HOPG or diamond–Si, model 1 (Figure 1a(i)) has a frequency response in the dispersion curve that saturates at a reduced frequency (*f*_1_/*f*_0_) that is lower than the measured reduced frequency. We note that the PtSi–PEO combination showed a high frequency response, which is unexpected for a soft material such as PEO. We attribute this to a very high elastic modulus measured a low penetration depths in PEO samples [26]. Model 3 (Figure 1a(iii))) in this case does not saturate as early, but the plateau in the dispersion curve translates into a wide variation in contact stiffness values assigned for very small changes in frequency. Thus, model 3 does not have sufficient accuracy for contact stiffness determination for these material systems. Model 2 (Figure 1a(ii)) slightly improves upon this issue, with the dispersion curve shifted more significantly to lower values of contact stiffness and a higher frequency plateau than model 1, such that improved accuracy in translating the measured cantilever frequency to a stiffness is possible. The additional benefit of model 2 over model 3 is that the model is much simpler and a friction coefficient, κ, between the tip/colloid and the substrate does not need to be assumed or calculated to generate the dispersion curve. However, as shown in Figure 1, the value of κ does not significantly change the positioning of the dispersion curve.

It has been suggested that careful selection of the cantilever stiffness is required when performing CR-AFM measurements [27]. Within the context of Figure 1, increasing the value of *k*_c_, while keeping all other material parameters constant, should shift the measured reduced frequency (*f_n_*/*f*_0_) left or to lower values, to a region of the dispersion curve where a more linear variation between frequency and stiffness is expected. In other words, with a very soft cantilever and a very hard sample, the saturated variation of the reduced frequency changes very little with contact stiffness, k*. We attempted to use cantilevers with a higher *k*_c_ value, ranging from 20 to 40 N·m^−1^, and perform the same analysis as done previously. As shown in Supporting Information File 1, Figure S2, the issue becomes that, with the stiffer cantilever, the magnitude of the resonance peak for the first normal mode, particularly when the tip contacts the surface, is much smaller than for the softer cantilevers. At this time, the base noise of our AFM system and electronic sampling of the deflection signal is too large to automate the fitting of the resonance peak with reasonable successful fits, limiting the application of our method to cantilevers having a lower spring constant.

With the frequency data translated to contact stiffness, the Derjaguin–Muller–Toporov (DMT), Johnson–Kendall–Roberts (JKR), and Carpick-Ogletree-Salmeron (COS) contact mechanics theories can be used to relate tip size, elastic modulus, and normal force. The relationship between contact stiffness, k*, and normal force for the DMT, JKR, and COS models are then given by Equation 5, Equation 6, and Equation 7, respectively [24,28]:


[5] $$k_{\text{DMT}}^{*}=2E^{*}{\left(\frac{R\left(3F+F_{\text{a}}\right)}{4E^{*}}\right)}^{1/3},$$

[6] $$k_{\text{JKR}}^{*}=2E^{*}{\left(\frac{3R(3F+2F_{\text{a}}+\sqrt{4FF_{\text{a}}+4F_{\text{a}}^{2}})}{2E^{*}}\right)}^{1/3},$$

**Figure 5:**
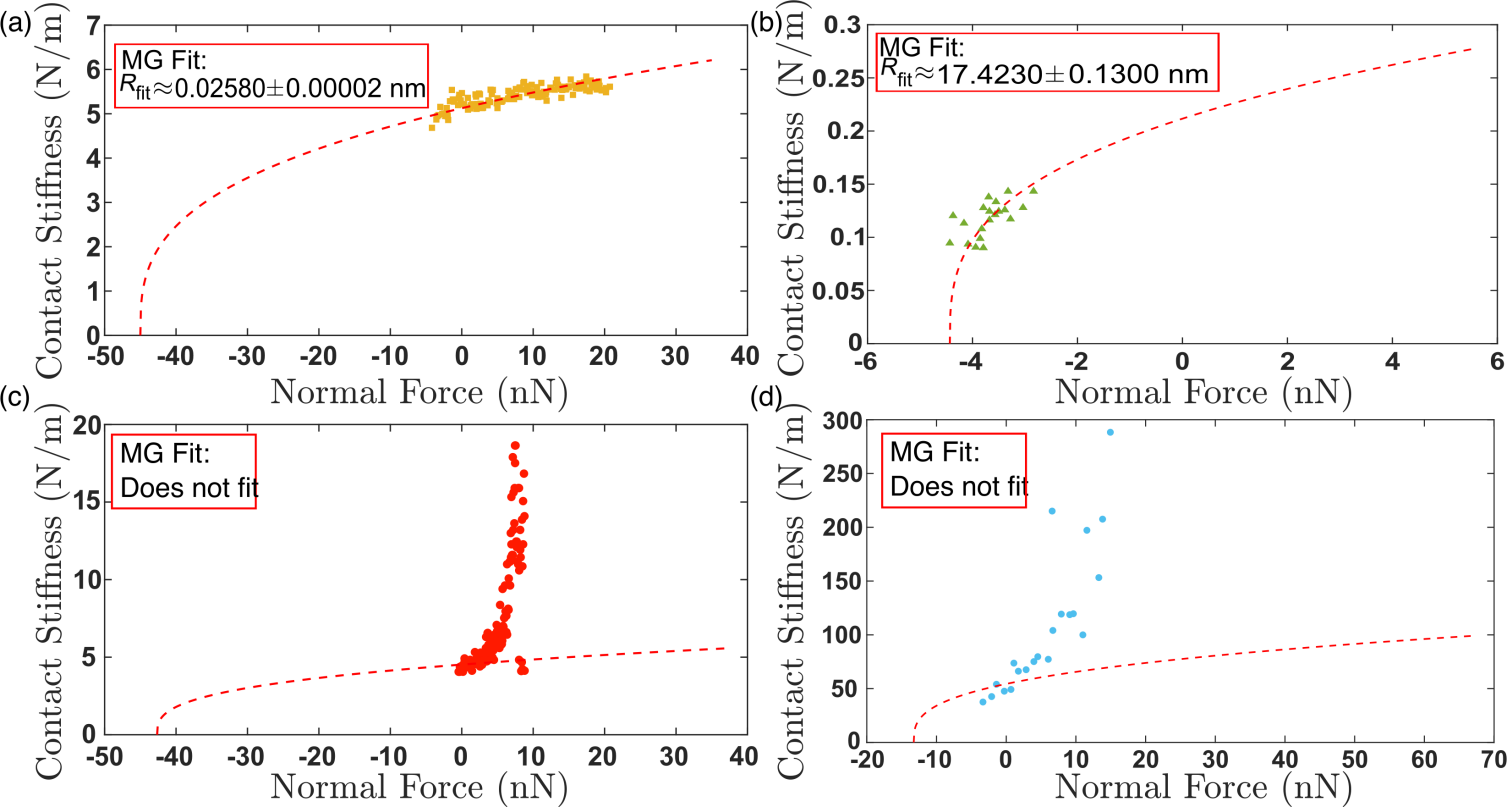
Contact stiffness versus normal force for (a) a silicon probe on HOPG sample (yellow squares) (b) a silicon probe on PDMS sample (green triangles), (c) a borosilicate glass colloid probe on a HOPG sample (red circles), and (d) a diamond-coated silicon on silicon sample (blue circles). A red dashed line in each figure shows a fit to the experimental data using Equation 7.

[7]

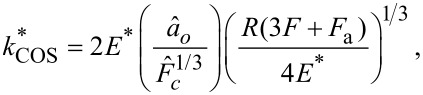



where *R* is the tip radius and *F*_a_ is the adhesive force. In Equation 7, we use the transition parameter λ to bridge the two contact streams. We then denote


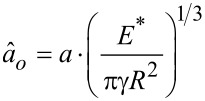


and


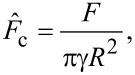


and γ is the work of adhesion, which can be calculated from the pull-off force in experiments. We calculated the Tabor parameter, μ*_T_*, and the transition parameter, λ, for each material pair, which are given in Table 2. Rather than fitting data with Tabor parameters smaller than 0.1 with the DMT model and greater than 5.0 with the JKR model [29], we use the COS model, which has been shown to more accurately fit contacts having material properties between the DMT and JKR extremes. The fits to the experimental data are provided in Figure 5. In each case, all materials for tip and substrate were pure amorphous/polycrystalline and, thus, had isotropic elastic moduli across the surface. Further, these materials were chosen as they are well characterized in the literature and often used in AFM experiments. Thus, rather than fitting the elastic modulus of the substrate, we took the elastic modulus values from literature for tip and substrate and fitted the radius of the probes using contact mechanics models. In many cases, the fits did not converge, so we used the best fit values near convergence and plotted the expected model variations for k* and normal force in Figure 5 with a red dashed line, with the experimental data overlayed in the graph. In each case, as stated previously, either the fits did not converge or yielded unphysical values for the tip radius. More specifically, Figure 5a and Figure 5b show converging fits to the experimental data, resulting in fits of 0.02580 ± 0.00002 nm and 17.42 ± 0.13 nm, respectively. Figure 5c and Figure 5d show results where the fits did not converge, with the experimental results clearly not following the predicted trend for contact stiffness by the MG model. In these cases, the radii estimated for the fits shown in Figure 5c and Figure 5d were 0.0011 and 0.0920 nm, respectively. This is a result of the very high stiffness of the contacting materials, which resulted in the reduced frequency having a value near the asymptote of the dispersion curve in models (ii) and (iii). Work by Killgore and Hurley identified that the analysis of stiff materials, or those having an elastic modulus greater than 10 GPa, with soft cantilevers such as those used in this experiment will not provide accurate results [30]. This finding is emphasized by the non-convergent fits observed in Figure 5c,d. However, the convergent fits in Figure 5a,b suggest that the CR-AFM models are valid, yet they result in unphysical values of the fit parameters. Therefore, our findings suggest that the CR-AFM models used are not applicable for a wider range of stiffness than previously thought. Finally, only by processing the cantilever deflection signal with STFT, such as here, or other time–frequency spectral analysis techniques, can sufficient temporal resolution of the oscillatory changes in the AFM cantilever within a single experiment be captured to perform such mechanical analysis and allow for the limits of the analytical models to be better validated.

**Table 2:** Tabor and transition parameters calculated for each material pairing.

Probe material	Sample material	Tabor parameter μ*_T_*	transition parameter λ

silicon	HOPG	0.4567	0.5284
diamond	silicon	0.1600	0.1851
glass colloid	HOPG	7.1923	8.3214
steel colloid	silicon	1.8955	2.1931
PtSi	PEO	3.1966	3.6985
silicon	PDMS	283.961	328.543

**Figure 6:**
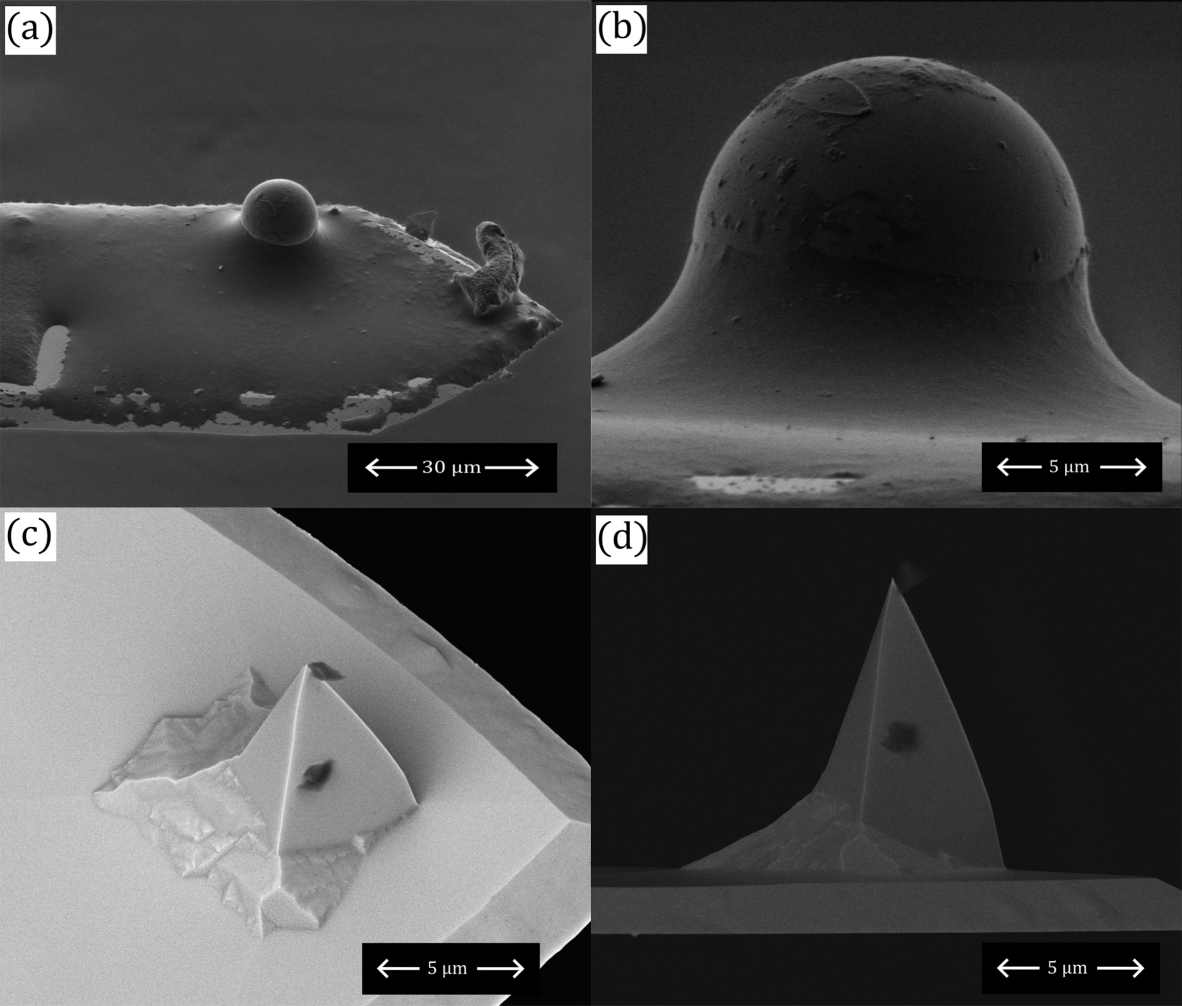
(a, b) Scanning electron microscopy images of the borosilicate glass colloid glued onto the tipless silicon cantilever. (c, d) Scanning electron microscopy images of the PtSi-coated AFM cantilever with integrated tip.

Figure 6 shows SEM images of two of the tips used in the study, that is, a borosilicate glass colloid glued onto a tipless silicon cantilever and a PtSi-coated silicon cantilever. In each of these cases, the tip radius was estimated to be much larger than what was fitted in Figure 5. While it is possible that, in particular with the colloidal probe, local surface roughness will yield a much smaller contact radius than the overall probe shape, it is still significantly larger than predicted by the models in Figure 5.

In summary, we have used longstanding analytical models to convert the measured variation in cantilever resonant frequency with applied normal force into contact stiffness. While the measurement process is very similar to what is typically done in CR-AFM studies, it becomes more clear as to why these studies normalize their results to a section or area of the surface with known mechanical properties: The analytical models that have been developed do not accurately describe the variation of cantilever frequency when the tip is pressed against the surface. At this time, no better models were developed to describe the link between cantilever frequency and contact stiffness, and we believe that normalization of the surface properties is the only method that makes it possible for experimentalists to provide some understanding of a quantitative value of the surface elastic modulus and other mechanical properties.

## Conclusion

High-data rate acquisition of the cantilever deflection signal from the photodiode of an AFM allows for the capture of the thermal motion of the AFM cantilever during a force-versus-distance measurement. STFT analysis was used to produce power spectra at regular time intervals during the experiments, with the frequency resolution varied to balance a faster time response of the cantilever’s oscillation parameters against the necessary frequency resolution to accurately fit the resonant peak of the first normal oscillation mode of the AFM cantilever. The resonance mode was fitted to a Lorentz peak to extract its center frequency and quality factor at each time point, providing similar information as to what is generated in a CR-AFM experiment. The cantilever resonant frequency was converted into contact stiffness using analytical models of cantilever vibrations, which could then be compared with contact mechanics models relating the applied normal force to contact stiffness. It was shown that those commercially available cantilevers, which provide enough signal for analysis in a standard AFM, push CR-AFM into a regime where small variations in frequency result in large variations of derived contact stiffness. This relationship between frequency and contact stiffness makes correlating experimental contact resonance data with contact stiffness, or other mechanical property assessments, very difficult. Thus, our findings show that, while high-fidelity data of the changing oscillatory behavior of AFM cantilevers can be obtained with high sampling rates and subsequent STFT analysis, quantitative analysis is not possible without measuring a calibration curve or normalizing data on a known material pair. These observations confirm why most CR-AFM studies report normalized data, despite providing information on the analytical models to convert frequency to contact stiffness in most cases, or only show qualitative frequency data. Further, we have been able to produce high-fidelity data that accurately captures the cantilever’s oscillation frequency and Q-factor over the course of the experiment, such that it can be compared directly with analytical models of cantilever oscillations and contact mechanics models, which had not been previously captured in the literature. Our study also shows that the current model used to describe CR-AFM experiments may not be complex enough to capture the physical experiment. However, choosing the cantilever stiffness knowing in advance what the expected material stiffness is may result in larger variations of contact stiffness with frequency than was captured in our study. Analysis of higher-order modes, having higher stiffnesses, may also improve the determination of contact stiffness from the measured resonant frequency.

## Supporting Information

File 1 Equations of motion of the cantilever dynamics models and additional experimental data.

## Data Availability

Data generated and analyzed during this study is available from the corresponding author upon reasonable request.
