# Supplementary material for: Mechanical property measurements enabled by short-term Fourier-transform of atomic force microscopy thermal deflection analysis
Source: Beilstein J Nanotechnol. 2025 Nov 6;16:1952–62. doi: 10.3762/bjnano.16.136 (PMC12599401; doi:10.3762/bjnano.16.136)
Supplement: File 1 — Equations of motion of the cantilever dynamics models and additional experimental data. [file Beilstein_J_Nanotechnol-16-1952-s001.pdf]

BEILSTEIN JOURNAL OF NANOTECHNOLOGY

Supporting Information

for

Mechanical property measurements enabled by short-term Fourier-transform of atomic force microscopy thermal deflection analysis

Thomas Mathias, Roland Bennewitz and Philip Egberts

Beilstein J. Nanotechnol. **2025,** *16,* 1952–1962. doi:10.3762/bjnano.16.136

Equations of motion of the cantilever dynamics models and additional experimental data

License and Terms: This is a supporting information file under the terms of the Creative Commons Attribution License (https://creativecommons.org/licenses/by/4.0). Please note that the reuse, redistribution and reproduction in particular requires that the author(s) and source are credited and that individual graphics may be subject to special legal provisions.
The license is subject to the *Beilstein Journal of Nanotechnology* terms and conditions: (https://www.beilstein-journals.org/bjnano/terms)

Equations of Dispersion Relations for Three Cantilever Models

Solution to model 1 dispersion

$$\sinh(k_nL)\cos(k_nL) - \sin(k_nL)\cosh(k_nL) = \frac{(k_nL)^3 k_\mathrm{c}}{3k^*}(1 + \cos(k_nL)\cosh(k_nL)) \quad \text{(S1)}$$

Solution to model 2 dispersion

$$\begin{aligned}
&-(\cosh(k_nL_1)\sin(k_nL_1) - \sinh(k_nL_1)\cos(k_nL_1))(1 + \cos(k_nL')\cosh(k_nL')) \\
&-(\cosh(k_nL')\sin(k_nL') - \sinh(k_nL')\cos(k_nL'))(1 - \cos(k_nL_1)\cosh(k_nL_1)) \\
&\qquad = 2k_n^3\frac{EI}{k^*}[1 + \cos(k_n(L_1 + L'))\cosh(k_n(L_1 + L'))] \quad \text{(S2)}
\end{aligned}$$

Solution to model 3 dispersion

$$\frac{k^*}{k_\mathrm{c}} = \frac{-B \pm \sqrt{B^2 - 4AC}}{6A} \quad \text{(S3)}$$

$$A = \left(\frac{\kappa}{k^*}\right)\left(\frac{h}{L_1}\right)^2 (1 - \cos(x)\cosh(xL_1))(1 + \cos(xL')\cosh(xL')) \quad \text{(S4)}$$

$$B = B_1 + B_2 + B_3 \quad \text{(S5)}$$

$$C = 2(xL_1)^4(1 + \cos(xL_1)\cosh(xL_1)) \quad \text{(S6)}$$

$$\begin{aligned}
B_1 &= \left(\frac{h}{L_1}\right)^2 (xL_1)^3 \left(\sin^2(\alpha) + \frac{\kappa}{k_\mathrm{c}}\cos^2(\alpha)\right) \\
&[(1 + \cos(xL')\cosh(xL'))(\sin(xL_1)\cosh(nL_1) + \cos(xL_1)\sinh(xL_1)) \\
&\qquad - (1 - \cos(nL_1)\cosh(nL_1))(\sin(xL')\cosh(xL') + \cos(xL')\sinh(xL'))] \quad \text{(S7)}
\end{aligned}$$

$$B_2 = 2\left(\frac{h}{L_1}\right)(xL_1)^2\left(\frac{\kappa}{k_c}\cos(\alpha)\sin(\alpha)\right)$$
$$[(1+\cos(xL')\cosh(xL'))(\sin(xL_1)\sinh(nL_1))$$
$$+(1-\cos(nL_1)\cosh(nL_1))(\sin(xL')\sinh(xL'))] \quad \text{(S8)}$$

$$B_3 = (xL_1)(\cos^2(\alpha)+\frac{\kappa}{k_c}\sin^2(\alpha))$$
$$[(1+\cos(xL')\cosh(xL'))(\sin(xL_1)\cosh(nL_1)-\cos(xL_1)\sinh(xL_1))$$
$$-(1-\cos(nL_1)\cosh(nL_1))(\sin(xL')\cosh(xL')-\cos(xL')\sinh(xL'))] \quad \text{(S9)}$$

$$G^* = \left(\frac{2-\nu_1}{G_1}+\frac{2-\nu_2}{G_2}\right)^{-1} \quad \text{(S10)}$$

$$G = \frac{1}{2}\left(\frac{E}{1+\nu}\right) \quad \text{(S11)}$$

$$\kappa = 8G^*a = \frac{8G^*k^*}{2E^*} = \frac{4k^*\left(\frac{2-\nu_1}{\frac{1}{2}\frac{E_1}{1+\nu_1}}\right)+\left(\frac{2-\nu_1}{\frac{1}{2}\frac{E_1}{1+\nu_1}}\right)}{E^*} \quad \text{(S12)}$$

Cantilever Frequency Power Spectrum

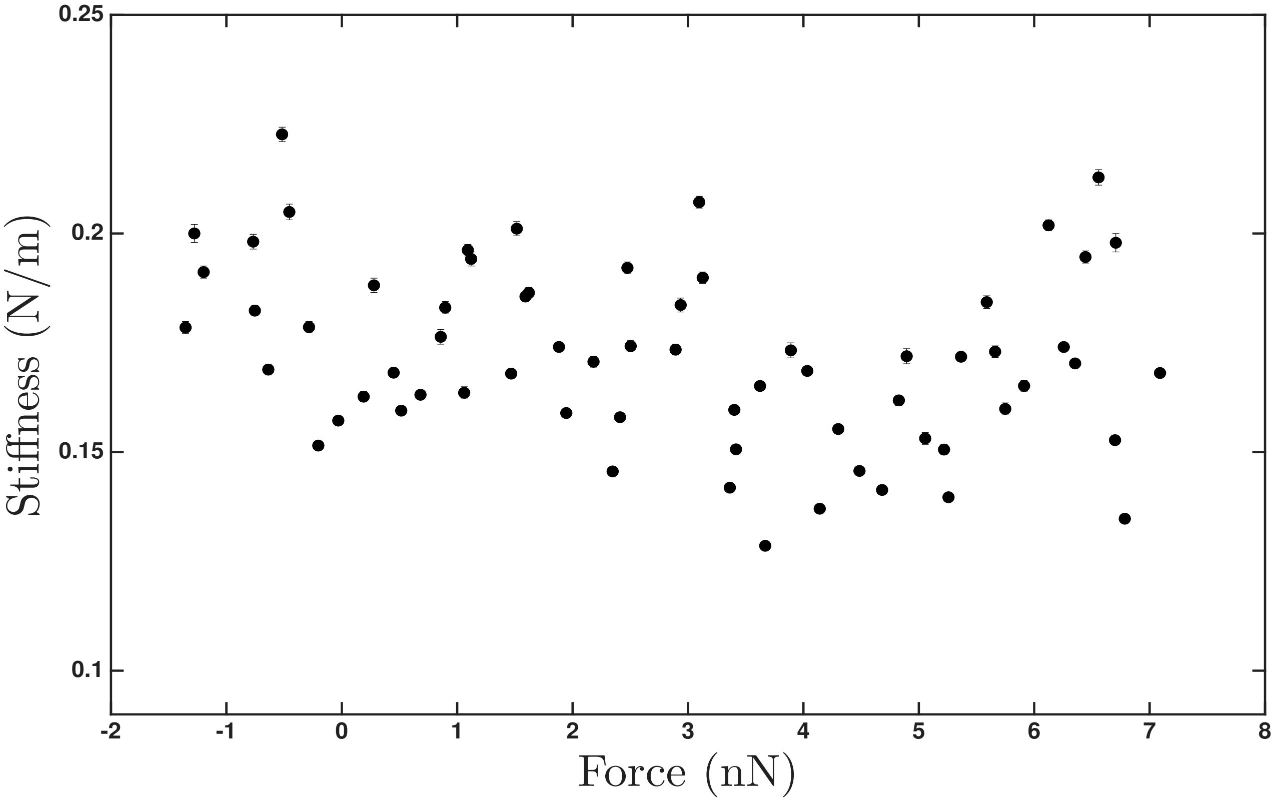

Figure S1: Stiffness versus normal force determined from fits of the first normal resonant mode peak in the power spectra of the contact portion of a force spectroscopy sweep.

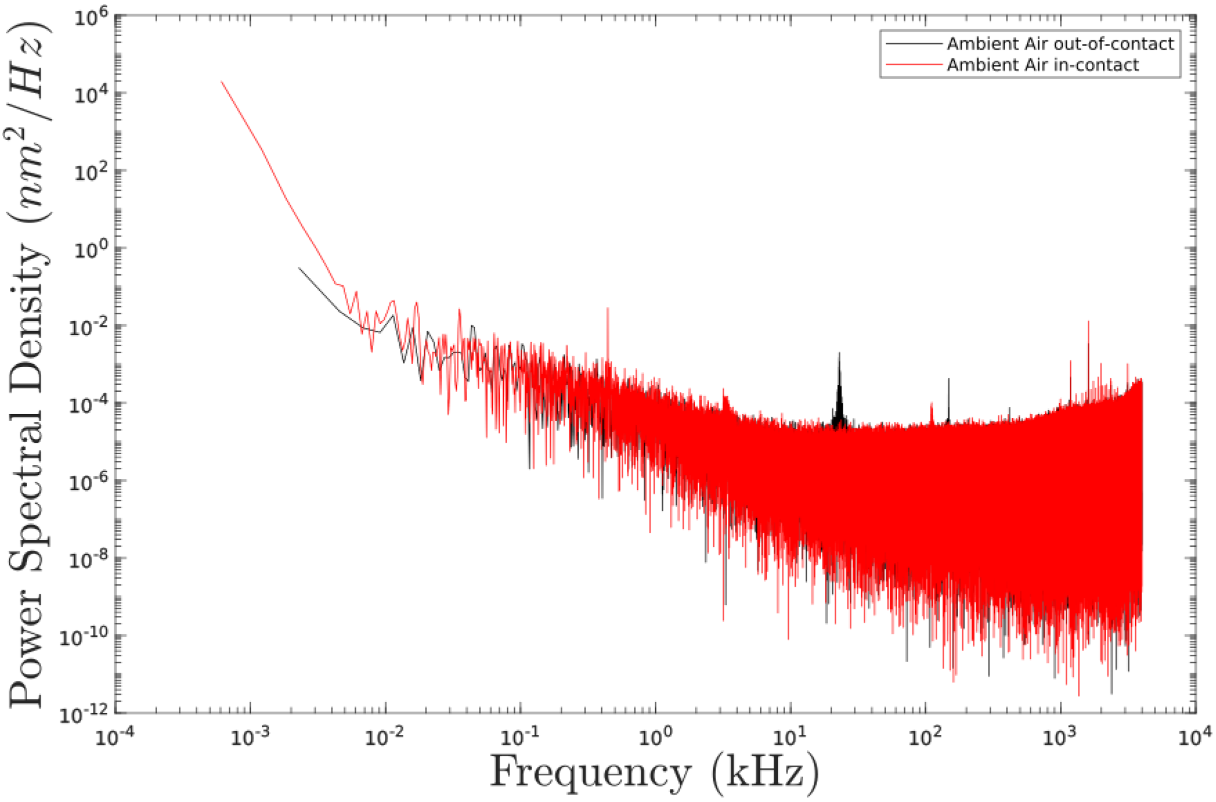

Figure S2: Fourier transform of the out-of-contact portion of a diamond coated probe having a spring constant between 20-40 N·m^{-1} on a silicon substrate. The out of contact portion is shown in black and the in-contact portion in red, highlighting the change in the resonant peak locations and shapes between these two stages of the measurement.

Elastic Modulus Determination of PEO

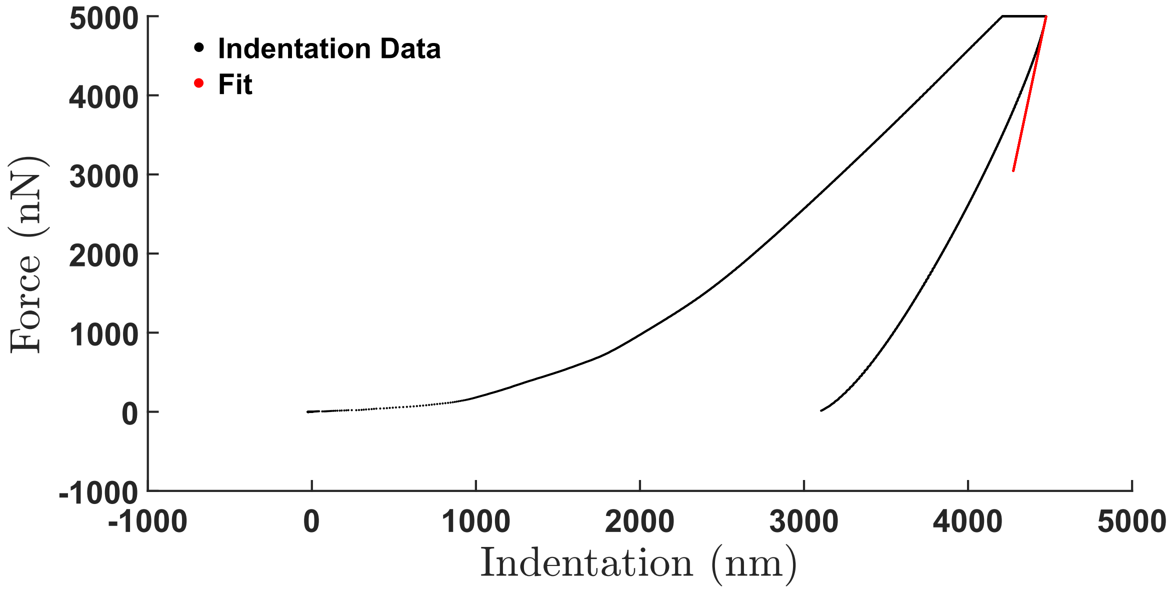

Figure S3: Example elastic unloading curves for the PEO sample obtained from nanoindentation experiments with a Berkovich indenter. Slope of curve in red is the linear fit used to determine the Young's modulus of the sample.
